# Supplementary material for: Urinary and Daily Assumption of Polyphenols and Hip-Fracture Risk: Results from the InCHIANTI Study
Source: Nutrients. 2022 Nov 10;14(22):4754. doi: 10.3390/nu14224754 (PMC9698374; doi:10.3390/nu14224754)
Supplement: Supplementary file 1 [file nutrients-14-04754-s001.zip › nutrients-1988691-supplementary.pdf]

Supplementary

Table S1: new drugs prescription in the follow-up and drugs prescribed at the baseline, in patient with compared to those subjects without Hip fracture.

	Hip	Fracture	
	No	Yes	
	781	36	p-value
**Bisfofonate**			0.06
New prescription	65 (8.3)	7 (19.4)	
At baseline	13 (1.7)	0 (0.0)	
**Glucocorticoids**			0.87
New prescription	39 (5.0)	2 (5.6)	
At baseline	13 (1.7)	1 (2.8)	
**Thyrosint**			0.88
New prescription	23 (2.9)	4 (11.1)	
At baseline	28 (3.6)	0 (0.0)	
**Anti-Parkinson**			0.83
New prescription	16 (2.1)	1 (2.8)	
At baseline	6 (0.8)	0 (0.0)	
**Insulin**			0.38
New prescription	8 (1.0)	1 (2.8)	
At baseline	8 (1.0)	1 (2.8)	
**Oral Hypoglycemic drugs**			0.67
New prescription	49 (6.3)	1 (2.8)	
At baseline	54 (6.9)	3 (8.3)	
**Vitamin supplementation**			0.54
New prescription	70 (9.0)	5 (13.9)	
At baseline	32 (4.1)	2 (5.6)	
**Diuretic**			0.84
New prescription	123 (15.8)	6 (16.7)	
At baseline	86 (11.0)	5 (13.9)	

Table S2: Diagnosis reported at baseline and during follow-up in patient with compared to those subjects without Hip fracture.

	Hip	Fracture	
	No	Yes	
	781	36	p-value
**Hypertension**			0.42
At Follow-up	97 (12.4)	5 (13.9)	
At baseline	502 (64.3)	26 (72.2)	
**Hepatic diseases**			0.72
At Follow-up	4 (0.5)	0 (0.0)	
At baseline	10 (1.3)	0 (0.0)	
**Stroke**			0.59
At Follow-up	37 (4.7)	3 (8.3)	
At baseline	44 (5.6)	1 (2.8)	
**Parkinson**			0.79
At Follow-up	29 (3.7)	1 (2.8)	
At baseline	8 (1.0)	0 (0.0)	
**Diabetes**			0.23
At Follow-up	37 (4.7)	0 (0.0)	
At baseline	99 (12.7)	7 (19.4)	
**Renal insufficiency**			0.50
At Follow-up	12 (1.5)	1 (2.8)	
At baseline	30 (3.8)	2 (5.6)	
